# A Synthetic Chloride Channel Restores Chloride Conductance in Human Cystic Fibrosis Epithelial Cells

**DOI:** 10.1371/journal.pone.0034694

**Published:** 2012-04-13

**Authors:** Bing Shen, Xiang Li, Fei Wang, Xiaoqiang Yao, Dan Yang

**Affiliations:** 1 Morningside Laboratory for Chemical Biology, Department of Chemistry, The University of Hong Kong, Hong Kong, China; 2 Department of Physiology, The Chinese University of Hong Kong, Shatin, Hong Kong, China; 3 Department of Physiology, Anhui Medical University, Hefei, China; Universidade Federal do Rio de Janeiro, Brazil

## Abstract

Mutations in the gene-encoding cystic fibrosis transmembrane conductance regulator (CFTR) cause defective transepithelial transport of chloride (Cl^−^) ions and fluid, thereby becoming responsible for the onset of cystic fibrosis (CF). One strategy to reduce the pathophysiology associated with CF is to increase Cl^−^ transport through alternative pathways. In this paper, we demonstrate that a small synthetic molecule which forms Cl^−^ channels to mediate Cl^−^ transport across lipid bilayer membranes is capable of restoring Cl^−^ permeability in human CF epithelial cells; as a result, it has the potential to become a lead compound for the treatment of human diseases associated with Cl^−^ channel dysfunction.

## Introduction

Chloride (Cl^−^) ion channels that mediate the flow of Cl^−^ ions through cell membranes play crucial roles in regulating a broad range of biological processes, including ion homeostasis, cell volume regulation, transepithelial transport and the regulation of electrical excitability [1]. The malfunctioning of Cl^−^ channels is implicated in many severe human diseases, most notably cystic fibrosis (CF) [2], [3]. In CF, mutations in the gene-encoding cystic fibrosis transmembrane conductance regulator (CFTR), a Cl^−^ channel located at the apical membranes of epithelial cells, causes defective transepithelial transport of Cl^−^ and fluid [4]–[7]. A general strategy for reducing the pathophysiology associated with CF mutations is to increase the Cl^−^ permeability of epithelial cells either through CFTR correctors or potentiators or via alternative pathways to compensate for the CFTR Cl^−^ channel defect. Great efforts have been made to develop gene transfer of the CFTR to epithelia [8]–[11] and therapeutic agents that can activate mutant CFTR genes [12], rescue mutant CFTR trafficking to the apical surface of epithelial cells [13], [14], suppress premature stop mutations located in the CFTR gene [15]–[17] or activate alternative natural Cl^−^ channels [7], [17]–[20]. Denufosol acting on the P2Y_2_ receptor opens up an alternative chloride channel resulting in Cl^−^ ion and liquid secretion, and partly compensates for effects caused by the mutant CFTR [19]. However, denufosol has recently failed phase III clinical trials. Therefore, the development of synthetic Cl^−^ channels [21]–[32] provides a brand new opportunity to enhance transepithelial Cl^−^ secretion for the treatment of CF.

To date, however, most reported synthetic ion channels have been characterized in artificial lipid bilayers, and biological applications of these synthetic Cl^−^ channels to increase the Cl^−^ permeability of living cells remain poorly explored [33]. Moreover, because most of these synthetic Cl^−^ channel-forming compounds have relatively complicated structures and high molecular weights [22]–[33], their application to drug discovery is restricted. In this study, we report on a small molecule that can form Cl^−^ channels in plasma membranes of living cells. This synthetic Cl^−^ channel is capable of increasing Cl^−^ conductance in human CF epithelial cells.

**Figure 1:**
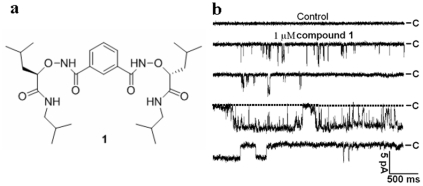
Chemical structure and single channel currents of compound 1 in HEK 293 cells. (**a**) Chemical structure of compound **1**. (**b**) Representative traces showing single channel currents with DMSO control (top trace) and the application of 1 µM compound **1** (lower four traces) at −60 mV in HEK 293 cells. “C” represents the base line in which the channel is in the close state.

We recently reported that the small molecule **1** (Figure 1, panel a) mediates Cl^−^ transport across lipid membranes [34]. The typical single-channel currents we observed for this molecule in giant liposomes confirmed the formation of ion channels [34]. Here, we address the electrophysiological properties and potential functions of this synthetic Cl^−^ channel in living cells.

## Results and Discussion

We first performed inside-out single channel recording, a patch clamp technique [35], to identify the formation of ion channels by compound **1** in HEK 293 cells, a widely used cell line in ion channel research. In the presence of 1 µM of compound **1**, the typical single-channel currents were indeed recorded in symmetric N-methylglucamine hydrochloride (NMDG-Cl) solutions (Figure 1, Panel b), indicating that the small molecule can self-assemble into functional ion channels in the cell membranes. Similar to single-channel recording with compound **1** in liposomes, various conductances were observed in the same or different patches, which was anticipated for molecules that self-assemble into ion channels of various sizes [22]–[33].

**Figure 2:**
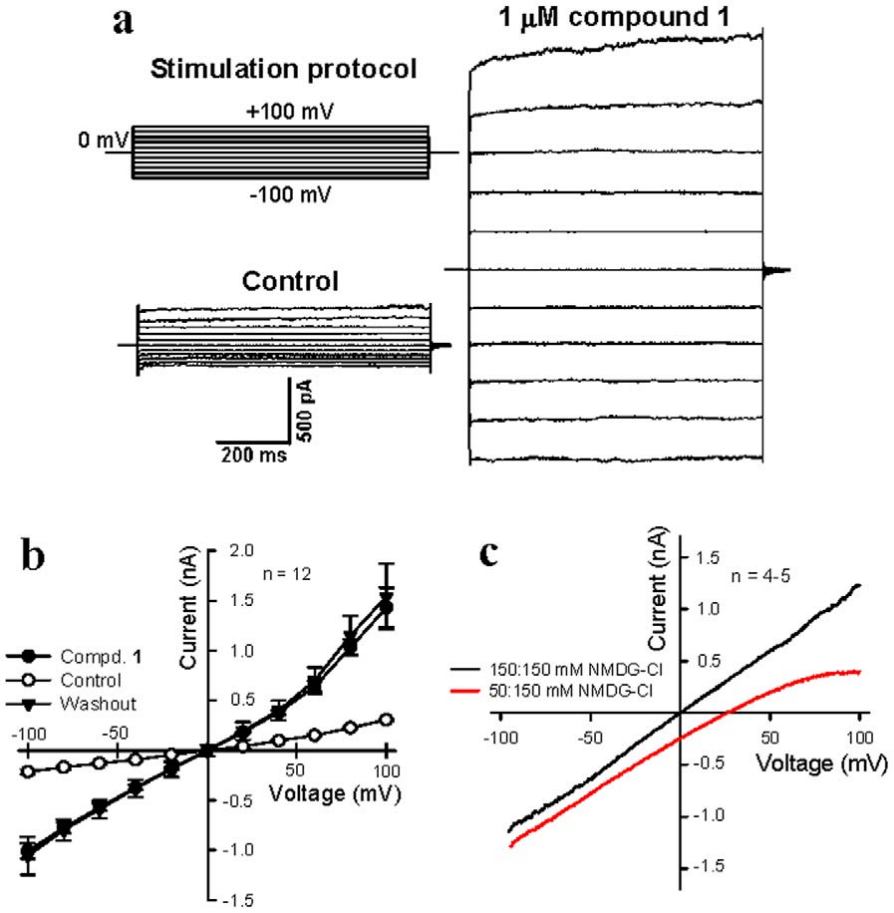
Electrophysiological properties of the ion channels formed by compound 1 in HEK 293 cells. (**a**) Representative traces showing whole-cell currents in HEK 293 cells. The upper panel shows the stimulation protocol for whole-cell recording, and the lower left panel shows DMSO control currents. The right panel shows whole-cell currents after the application of 1 µM compound **1**. (**b**) Current–voltage relationships obtained in the absence (○) and presence (•) of 1 µM compound 1 and after washout (▴) with the control bath solution for 30 min in HEK 293 cells. (**c**) Representative traces showing the whole-cell ramp currents recorded in symmetric 150 mM NMDG-Cl (black trace) and asymmetric NMDG-Cl with 150 mM in internal and 50 mM in external solutions (red trace). All data are means ± s.e. n = 5–6.

We then used the whole-cell configuration of patch clamp technique to examine the electrophysiological properties of the ionic currents induced by compound **1** in HEK 293 cells. Whole-cell patch clamp recording was first performed in standard intracellular and extracellular solutions (see Methods). While the vehicle (0.1% DMSO) had no effect on the whole-cell currents (Figure S1), compound **1**, even at a low concentration of 1 µM, induced a large current increase relative to that of the control (Figure 2, Panels a and b). To confirm that the increase in whole-cell currents was caused by the formation of synthetic Cl^−^ channels, we next recorded the whole-cell currents in symmetric bath and pipette solutions of NMDG-Cl. As expected, there was no obvious difference in the whole-cell currents elicited by compound **1** when the cations (Na^+^, Cs^+^ and Mg^2+^) used in the standard bath and pipette solutions were replaced by the larger NMDG cation (Figure 2, Panels b and c), which is known to be impermeable through most natural cation channels. However, when the NMDG-Cl bath solution was changed from symmetric 150 mM to asymmetric 50 mM, the outward current decreased to a remarkable extent and the cell reverse potential (*E*_rev_) shifted from 0 to 26.99±1.38 (n = 5) mV, which is quite close to the value of the Cl^−^ reverse potential (*E*_Cl_) (27.76 mV) calculated from the Nernst equation. Taken together, these results indicate that the ion channels formed by compound **1** are permeable to Cl^−^ ions only, and not to NMDG ions, and the increased whole-cell currents observed in previous experiments can therefore be attributed to the Cl^−^ currents induced by synthetic Cl^−^ channels rather than to a non-specific membrane leak.

**Figure 3:**
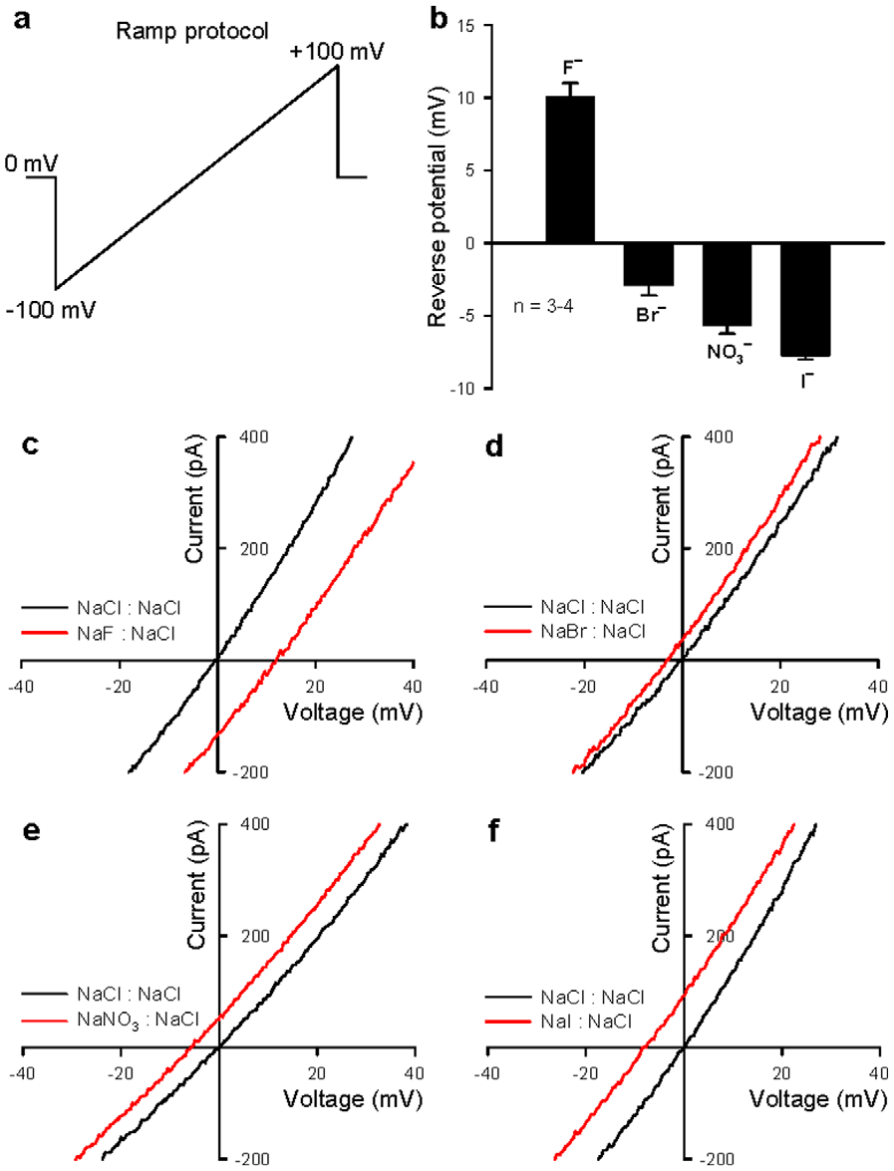
The anion selectivity of synthetic Cl^−^ channel 1 in HEK 293 cells. (**a**) The ramp stimulation protocol for whole-cell recording. (**b**) The data summary for reverse potential changes when external solutions were changed from symmetric 150 mM Cl^−^ to 150 mM F^−^, Br^−^, NO_3_^−^ and I^−^, respectively. All data are means ± s.e. n = 3–4. (c–f) Zoomed representative traces showing changes in reverse potential when external solutions were changed from 150 mM Cl^−^ (black traces) to 150 mM (**c**) F^−^, (**d**) Br^−^, (**e**) NO_3_^−^ and (**f**) I^−^ (red traces), respectively.

We next investigated the anion selectivity of this synthetic Cl^−^ channel by comparing cell reverse potentials when replacing the extracellular Cl^−^ ions with equimolar F^−^, Br^−^, I^−^, and NO_3_^−^ ions. The relative permeability ratios against Cl^−^ (F^−^ ∶ Cl^−^ ∶ Br^−^ ∶ I^−^ ∶ NO_3_^−^) were determined to be 0.673 ∶ 1 ∶ 1.121 ∶ 1.251 ∶ 1.358 (Figure 3), i.e., the anion transport activity of the synthetic Cl^−^ channel follows the sequence F^−^<Cl^−^<Br^−^<I^−^<NO_3_^−^. Meanwhile, the same anion over cation selectivity and relative transport activity toward various anions in patch-clamp studies have also been observed in fluorescence assays performed in liposomes (Figure S2, Panels a and b). Moreover, in our effort to search for potential inhibitors of this synthetic Cl^−^ channel, we examined the effects of a variety of commonly used blockers of natural Cl^−^ channels. Unfortunately, all the inhibitors tested (DIDS, DPC, NFA, SITS and NPPB) failed to block the whole-cell currents induced by compound **1** (Figure S3). This result was not unexpected given that the synthetic Cl^−^ channel may have very different structural features from those of natural ones.

**Figure 4:**
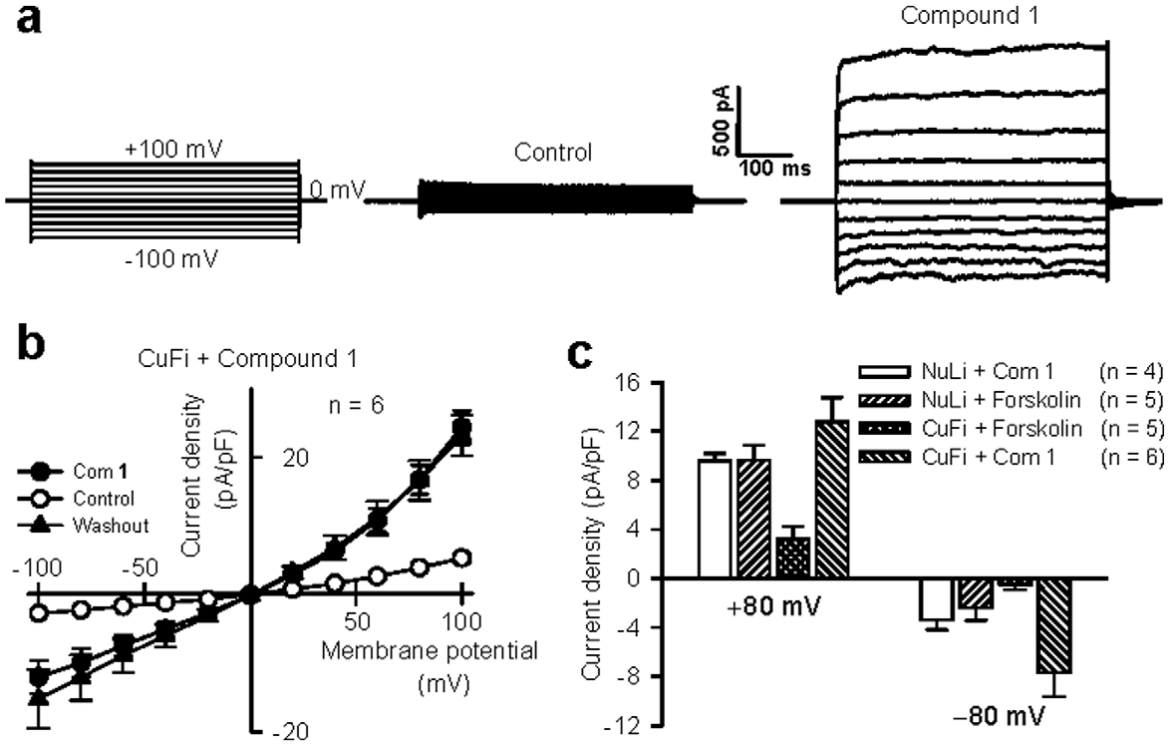
Synthetic Cl^−^ channel formed by compound 1 restored cell Cl^−^ permeability in CF cells. (**a**) Representative traces showing whole-cell currents in CF cells. The left panel shows the stimulation protocol for whole-cell recording, and the middle panel shows DMSO control currents. The right panel shows whole-cell currents after the application of 1 µM compound **1**. (**b**) The current–voltage relationships obtained in the absence (○) and presence (•) of 1 µM compound **1** and after washout (▴) with the control bath solution for 30 min in CuFi-8 cells. (**c**) Summary of whole-cell currents at ±80 mV induced by forskolin (2 µM) and compound **1** (1 µM) in CuFi-8 and NuLi-1 cells. All data are means ± s.e. n = 5–6.

To determine the potential of using compound **1** in the treatment of CF, the final step of our analysis involved investigating whether compound **1** could be incorporated into the plasma membranes of CF cells and, thereby, function as a Cl^−^ channel to increase the Cl^−^ permeability of these cells. We recorded the whole-cell currents induced by compound **1** in CuFi-8 and NuLi-1 cells, [34] which were derived from the human bronchial epithelia of a patient with CF (CuFi-8, homozygous CFTR ΔF508 mutant) and a subject without CF (NuLi-1, wild type CFTR), respectively. The application of compound **1** induced a gradual increase in the whole-cell currents in the first few minutes, after which the currents became stable for 15 minutes. The currents were persistent even after washing out the cells with control buffer for 30 minutes (Figure 4, Panel b). Interestingly, even at a low concentration of 1 µM, compound **1** induced very large whole-cell currents relative to the basal currents (Figure 4, Panels a and b). The whole-cell currents of the CF cells treated with compound **1** displayed a slightly voltage-dependent current-voltage relationship similar to that seen in HEK 293 cells. Furthermore, panel c of Figure 4 reveals that the addition of forskolin (2 µM), an agonist of cAMP, led to a large increase in the Cl^−^ currents of the normal human airway epithelial cells (NuLi-1), whereas the CF cells (CuFi-8) exhibited little response to forskolin. In contrast, the addition of 1 µM compound **1** resulted in an even larger increase in the whole-cell currents of the CuFi-8 cells than that recorded in those of the NuLi-1 cells treated with 2 µM forskolin, indicating that compound **1** is capable of increasing Cl^−^ conductance in these CF cells via a novel pathway. Similarly, compound **1** also increased Cl^−^ conductance in NuLi-1 cells. The compound **1**-induced Cl^−^ current was a little smaller than that recorded in CuFi-8 cells. Because compound **1** is generated from an α-aminoxy acid unit, in considering the possibility of applying compound **1** in future, we also examined its toxicity in, for example, immune response in animals. The data showed that compound 1 did not induce mouse immune response, especially in lung tissue (Figures S4 and S5, Methods S1). However, even though compound **1** exhibited a very good ability to restore the Cl^−^ permeability of CF airway epithelial cells and had low toxicity in animals, we still cannot conclude that it can be used to treat CF in practice. Further experiments based on a CF animal model should provide better evidence confirming the therapeutic potential of compound **1**. At this stage, we provide a preliminary report of our findings.

In conclusion, we have demonstrated that compound **1** forms functional Cl^−^ channels not only in lipid bilayer membranes of liposomes, but also in those of live cells, as revealed by both single-channel and whole-cell patch clamp experiments. More importantly, compound **1** is capable of increasing the Cl^−^ permeability of CF airway epithelial cells with defects in their native CFTR Cl^−^ channels. In light of these observations, this compound may have therapeutic potential for the treatment of CF lung disease or for treating other diseases that might benefit from increased Cl^−^ transport.

## Methods

### Cell culture

The HEK 293 cell line obtained from the American Type Culture Collection was cultured in DMEM supplemented with 10% FBS, 100 IU/ml penicillin G and 0.1 mg/ml streptomycin. NuLi-1 and CuFi-8 cells, a generous gift from Prof. Joseph Zabner (University of Iowa, Iowa City, IA), were derived from the human bronchial epithelia of a patient without CF (NuLi-1, WT CFTR) and a subject with CF (CuFi-8, homozygous CFTR ΔF508 mutant), respectively. These cell lines were grown on human placental collagen type VI (Sigma)–coated flasks in BEGM (Cambrex Bio Science, Walkersville, MD) medium, which includes BEGM basal medium and eight SingleQuots of supplements. Cells were grown at 37°C in a 5% CO_2_ humidified incubator.

### Electrophysiology

The single channel current was recorded using an EPC 9 patch clamp amplifier (HEKA Elektronik, Lambrecht/Pfalz, Germany) in voltage-clamp mode, controlled by Pulse/PulseFit 8.7 software (HEKA) with inside-out patch configuration. Patch pipettes (resistance, 10–20 MΩ) were filled with internal pipette solution containing 150 mM NMDG-Cl and 10 mM HEPES (pH 7.2). The bath was composed of symmetric 150 mM NMDG-Cl and 10 mM HEPES (pH 7.2) with 200 µM diphenylamine-2-carboxylic acid that blocks native Cl^−^ channels. After the inside-out recording was achieved without any channel opening, 1 µM compound **1** was perfused into the bath. The single channel current was recorded continuously and sampled at 4 kHz, and was typically low-pass filtered at 0.4 kHz with an 8-pole Bessel filter.

In the whole-cell current recording of the HEK 293, NuLi-1 and CuFi-8 cells in the standard bath (NaCl 140, CsCl 5, CaCl_2_ 1, MgCl_2_ 1 and HEPES 10 in mM, pH 7.4 with CsOH) and pipette (CsCl 140, MgCl_2_ 1, HEPES 10, EGTA 5 and Na_2_ATP 5 in mM, pH 7.2 with CsOH) solutions, the cells were held at 0 mV and voltage steps ranging from −100 to +100 mV were applied for 800 ms in 20-mV step increments. In these experiments, the native K^+^ currents were eliminated by using K^+^-free intra- and extracellular solutions containing Cs^+^, and any low threshold native Ca^2+^ or Na^+^ channels were inactivated by using a holding potential of 0 mV. Internal free Ca^2+^ was chelated by EGTA in pipette solution to prevent Ca^2+^-dependent channels from being activated. Changes in the whole-cell currents were detected from the same cells before and after exposure to the bath solution containing compound **1** (1 µM, 1 mM stock in DMSO) or forskolin (2 µM, 2 mM stock in DMSO). Unless indicated otherwise, all control data compared with compound **1** were from the 0.1% DMSO treatment. In ion selectivity experiments, the symmetric solution was first used in both intra- and extracellular sides. The cells were held at 0 mV and whole-cell currents were recorded with a ramp from −100 mV to +100 mV for 500 ms. Liquid junction potential was calculated by pClampfit 9.0 software (Axon Instruments, Inc., Sunnyvale, USA) and the corresponding reverse potential was determined. After observing a stable whole-cell current induced by the application of 1 µM compound **1**, the bath solution was replaced by isotonic (adjusted by sucrose) desired extracellular solution also containing 1 µM compound **1**. In these experiments, all bath solutions containing 200 µM diphenylamine-2-carboxylic acid were used to suppress native Cl^−^ channels other than those conducted to investigate the effect of Cl^−^ channel inhibitors. All patch-clamp experiments were performed at room temperature (22–25°C).

### pH-stat HPTS assay

Typically, 100 µL of the HPTS-loaded liposomes (stock solution) was suspended in 1.9 mL of the corresponding buffer and placed into a fluorimetric cell. HPTS emission at 510 nm was monitored during simultaneous excitation at wavelengths of 403 and 460 nm. During each experiment, 20 µL of a 1 mM THF solution of compound **1** (10 µM final) was added through an injection port, followed by the injection of 20 µL of 0.5 M aqueous NaOH. The addition of NaOH caused in increase of ca. 1 pH unit in the extravesicular buffer. The maximal change in dye emission was obtained at the end of each experiment after lysis of the liposomes with a detergent (40 µL of 5% aqueous Triton X-100). The final transport trace was obtained from the ratio of the emission intensities monitored at 460 and 403 nm and was normalized to 100% of transport.

## Supporting Information

Figure S1 **Current–voltage relationships obtained in the absence (•) and presence (○) of 0.1% DMSO in HEK 293 cells.**
(PDF)

Figure S2 **pH-Stat ion transport assays illustrating the ion selectivity of compound 1.** All experiments employed suspensions of EYPC liposomes containing the pH-sensitive dye HPTS in a HEPES buffer. The intravesicular solutions were 10 mM HEPES (pH 6.8) and 100 mM NaCl and extravesicular solutions were 10 mM HEPES (pH 6.8) and 100 mM MCl (M = Na^+^, K^+^ and Cs^+^) in **a**. Both the intra- and extravesicular solutions contained 10 mM HEPES (pH 6.8) and 100 or 75 mM Na_n_X (X = Cl^−^, Br^−^, NO_3_^−^, SO_4_^2−^) in **b**. At t = 100 s, a THF solution (20 µL) of the testing compound at 10 µM final concentration was added to the extravesicular solution; 0.5 M NaOH solution (20 µL) was then added. At t = 700 s, 5% Triton X-100 (40 µL) was added to lyse the liposomes.
(PDF)

Figure S3 **The data summary of 1 µM compound 1-increased whole-cell current densities (pA/pF) in the absence (control) and presence of 100 µM 4,4′-dithiocyanatostilbene -2,2′- disulfonic acid (DIDS), 100 µM 4-acetamido-4′-isothiocyanostilbene-2,2′-disulfonic acid (SITS), 200 µM diphenylamine-2-carboxylic acid (DPC), 100 µM 5-nitro-2-(3-phenylpropylamino)-benzoic acid (NPPB), and 100 µM niflumic acid (NFA), respectively, at ±80 mV in HEK 293 cells.** The cells were pretreated with the inhibitors, respectively, for 10 min before the application of 1 µM compound 1. All data are mean ± s.e. n = 3–8, *P*>0.05 compared to control group.
(PDF)

Figure S4 **The data summary of serum TNF-α concentration of mice in the first 24 hrs after intraperitoneal injection with DMSO, lipopolysaccharide (LPS) or compound 1 (Comp 1).** DMSO is a negative control because it is the solvent of compound 1. LPS is a well-known endotoxin and capably of eliciting strong immune responses in animals. Here, LPS is a positive control. All data are mean ± s.e. n = 4–6 mice, *P*>0.05 compared to DMSO group. *P*<0.01 compared to DMSO group.
(PDF)

Figure S5 **The H & E staining images of mice lung sections in the first 24 hrs after intraperitoneal injection with DMSO, lipopolysaccharide (LPS) or compound 1 (Comp 1).** DMSO is a negative control because it is the solvent of compound 1. LPS is a well-known endotoxin and capably of eliciting strong immune responses in animals. Here, LPS is a positive control. The images show inflammatory cells (labeled by blue arrow) infiltration in LPS treatment, but no inflammatory cells can be found in DMSO and compound 1 treatment.
(PDF)

Methods S1 **Immune response test in mouse.**
(DOC)
