# Correction: A Synthetic Chloride Channel Restores Chloride Conductance in Human Cystic Fibrosis Epithelial Cells

**DOI:** 10.1371/annotation/024a8dbe-bc8c-40d1-8c0a-912bc94d04ef

**Published:** 2012-07-03

**Authors:** Bing Shen, Xiang Li, Fei Wang, Xiaoqiang Yao, Dan Yang

The following information is missing from the Funding section: Grant HKU8/CRF/10 from The University of Hong Kong and the Hong Kong Research Grants Council. 

