# Supplementary figures and images for: A Synthetic Chloride Channel Restores Chloride Conductance in Human Cystic Fibrosis Epithelial Cells
Source: PLoS One. 2012 Apr 13;7(4):e34694. doi: 10.1371/journal.pone.0034694 (PMC3326041; doi:10.1371/journal.pone.0034694)

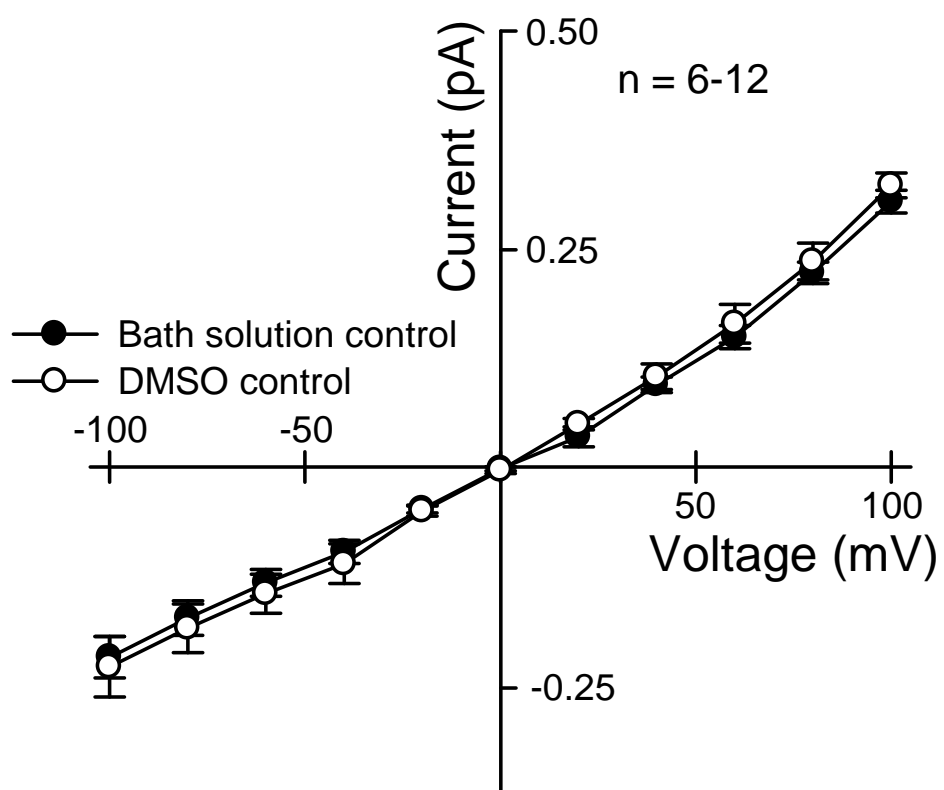

Supplement: Figure S1 — Current–voltage relationships obtained in the absence (•) and presence (○) of 0.1% DMSO in HEK 293 cells. (PDF) [file pone.0034694.s001.pdf]

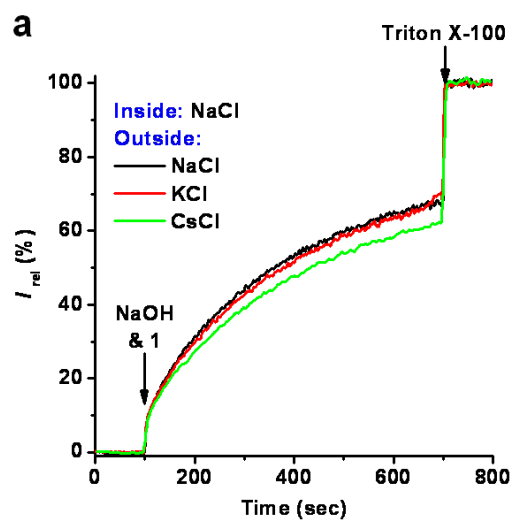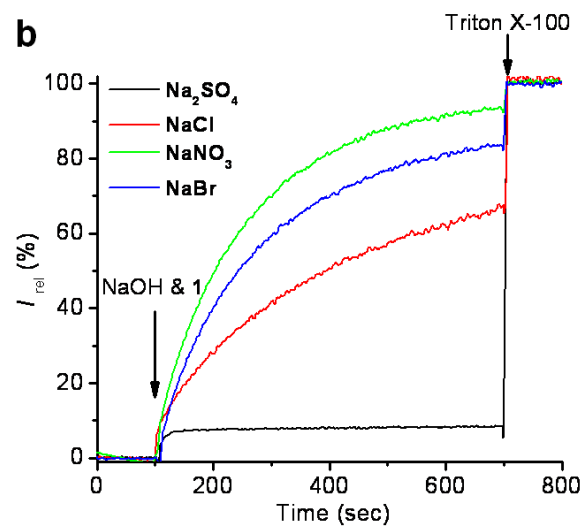

Supplement: Figure S2 — pH-Stat ion transport assays illustrating the ion selectivity of compound 1. All experiments employed suspensions of EYPC liposomes containing the pH-sensitive dye HPTS in a HEPES buffer. The intravesicular solutions were 10 mM HEPES (pH 6.8) and 100 mM NaCl and extravesicular solutions were 10 mM HEPES (pH 6.8) and 100 mM MCl (M = Na+, K+ and Cs+) in a. Both the intra- and extravesicular solutions contained 10 mM HEPES (pH 6.8) and 100 or 75 mM NanX (X = Cl−, Br−, NO3 −, SO4 2−) in b. At t = 100 s, a THF solution (20 µL) of the testing compound at 10 µM final concentration was added to the extravesicular solution; 0.5 M NaOH solution (20 µL) was then added. At t = 700 s, 5% Triton X-100 (40 µL) was added to lyse the liposomes. (PDF) [file pone.0034694.s002.pdf]

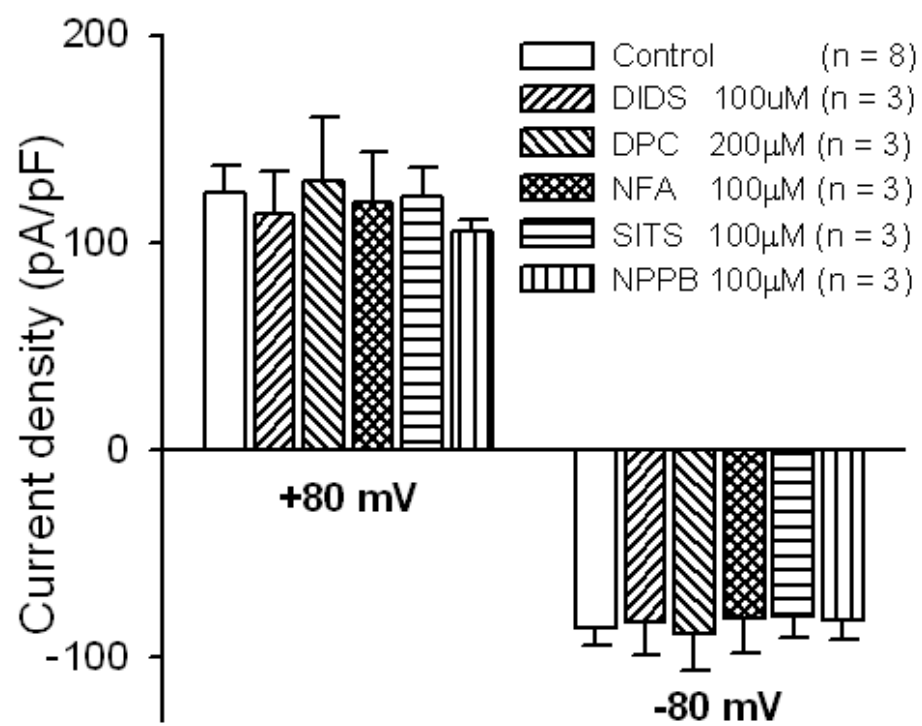

Supplement: Figure S3 — The data summary of 1 µM compound 1-increased whole-cell current densities (pA/pF) in the absence (control) and presence of 100 µM 4,4′-dithiocyanatostilbene -2,2′- disulfonic acid (DIDS), 100 µM 4-acetamido-4′-isothiocyanostilbene-2,2′-disulfonic acid (SITS), 200 µM diphenylamine-2-carboxylic acid (DPC), 100 µM 5-nitro-2-(3-phenylpropylamino)-benzoic acid (NPPB), and 100 µM niflumic acid (NFA), respectively, at ±80 mV in HEK 293 cells. The cells were pretreated with the inhibitors, respectively, for 10 min before the application of 1 µM compound 1. All data are mean ± s.e. n = 3–8, P>0.05 compared to control group. (PDF) [file pone.0034694.s003.pdf]

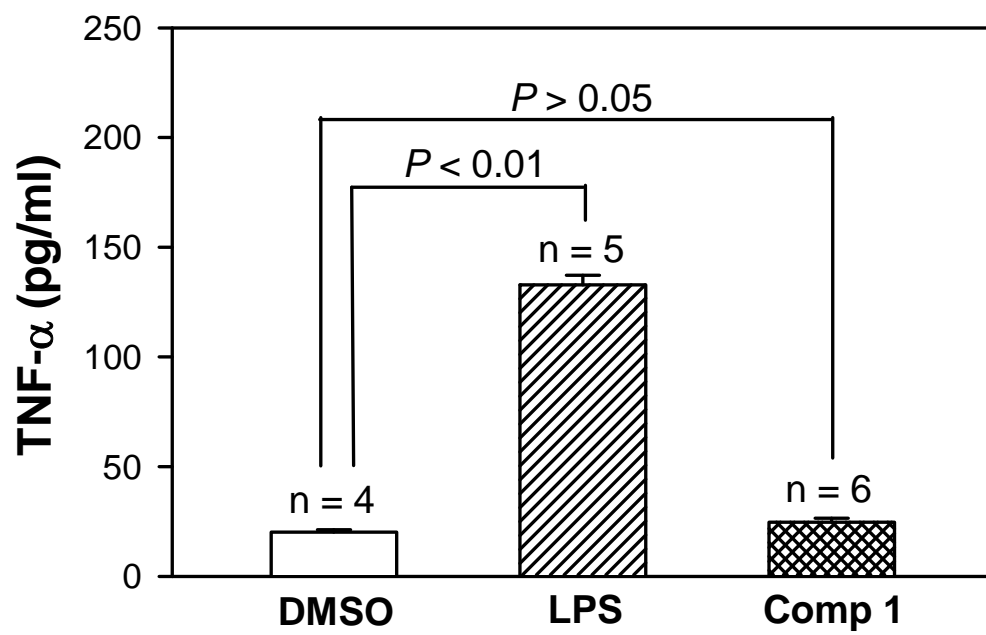

Supplement: Figure S4 — The data summary of serum TNF-α concentration of mice in the first 24 hrs after intraperitoneal injection with DMSO, lipopolysaccharide (LPS) or compound 1 (Comp 1). DMSO is a negative control because it is the solvent of compound 1. LPS is a well-known endotoxin and capably of eliciting strong immune responses in animals. Here, LPS is a positive control. All data are mean ± s.e. n = 4–6 mice, P>0.05 compared to DMSO group. P<0.01 compared to DMSO group. (PDF) [file pone.0034694.s004.pdf]

LPS

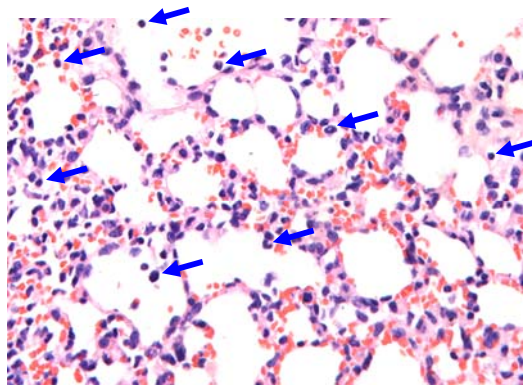

DMSO

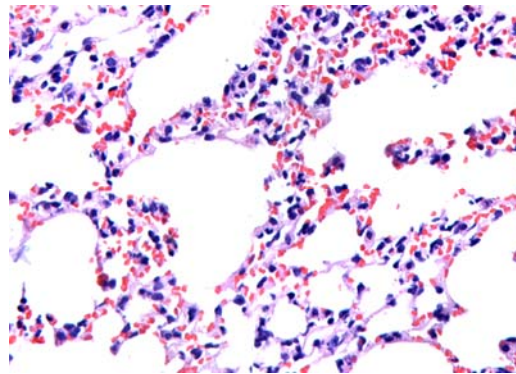

Comp 1

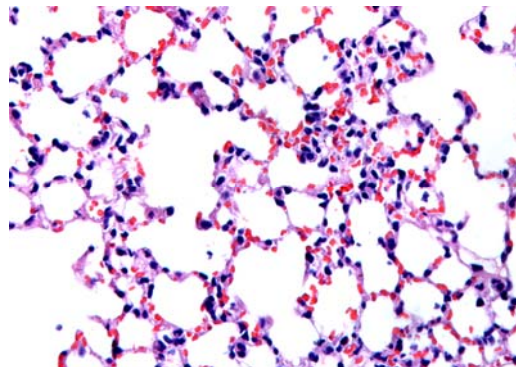

Supplement: Figure S5 — The H & E staining images of mice lung sections in the first 24 hrs after intraperitoneal injection with DMSO, lipopolysaccharide (LPS) or compound 1 (Comp 1). DMSO is a negative control because it is the solvent of compound 1. LPS is a well-known endotoxin and capably of eliciting strong immune responses in animals. Here, LPS is a positive control. The images show inflammatory cells (labeled by blue arrow) infiltration in LPS treatment, but no inflammatory cells can be found in DMSO and compound 1 treatment. (PDF) [file pone.0034694.s005.pdf]
