# Supplementary material for: A Synthetic Chloride Channel Restores Chloride Conductance in Human Cystic Fibrosis Epithelial Cells
Source: PLoS One. 2012 Apr 13;7(4):e34694. doi: 10.1371/journal.pone.0034694 (PMC3326041; doi:10.1371/journal.pone.0034694)
Supplement: Methods S1 — Immune response test in mouse. (DOC) [file pone.0034694.s006.doc]

# Supporting Information

**A Synthetic Chloride Channel Restores Chloride Conductance in Human Cystic Fibrosis Epithelial Cells**

# Bing Shen^2,3,4^, Xiang Li^1,4^, Fei Wang^3^, Xiaoqiang Yao^2^*, Dan Yang^1^*

^1^Morningside Laboratory for Chemical Biology, Department of Chemistry, The University of Hong Kong, Pokfulam Road, Hong Kong, China

^2^Department of Physiology, The Chinese University of Hong Kong, Shatin, N.T., Hong Kong, China

^3^Department of Physiology, Anhui Medical University, Hefei, China

^4^These authors contributed equally to this work.

# *, Address correspondence to Xiaoqiang Yao, Ph.D and Dan Yang:

# yao2068@cuhk.edu.hk and yangdan@hku.hk

# Supporting Methods:

**Immune response test in mouse:** All animal experiments complied with national guidelines and were approved by the Institutional Animal Care and Use Committee. Mice with 6-8 weeks age were anaesthetized with pentobarbital sodium. DMSO (same volume as compound **1**), lipopolysaccharide (LPS, 5 mg/kg) or compound **1** (5 mg/kg) was intraperitoneal injected into mice respectively. After 24 hrs, mice were killed by CO_2_. The blood samples were collected and the lung tissues were isolated. The concentration of serum TNF-α was examined with ELISA kit (Shanhai Westang Bio-Tech Co. Ltd.) according to manual. Mice lung tissues were fixed with 3.7% formaldehyde overnight in 4 degree and after following washout, the tissues were embedded with wax. Then the tissues were sectioned and H & E staining was performed. The sections were viewed under the microscope with 40 X objective lens.
